# EBV BART microRNA Profiles and Host Gene Links in Gastric Cancer

**DOI:** 10.3390/v18030329

**Published:** 2026-03-07

**Authors:** Esra Dirimtekin, D. Alwyn Dart, Pinar Uysal-Onganer

**Affiliations:** 1 Department of Medical Genetics, School of Medicine, Marmara University, 34854 Istanbul, Turkey; esradirimtekin@gmail.com; 2 UCL Cancer Institute, University College London, Paul O’Gorman Building, 72 Huntley Street, London WC1E 6DD, UK; a.dart@ucl.ac.uk; 3 Cancer Mechanisms and Biomarkers Research Group, School of Life Sciences, University of Westminster, London W1W 6UW, UK

**Keywords:** Epstein–Barr virus, gastric cancer, viral microRNAs, BART miRNAs, tumour microenvironment

## Abstract

Epstein–Barr virus (EBV), a ubiquitous human herpesvirus infecting over 90% of the adult population, is causally associated with a distinct molecular subtype of gastric cancer (GC). A key mechanism by which EBV influences tumour biology is the expression of viral microRNAs (miR/miRNA) encoded within the BamHI-A rightward transcript (BART) region, although inter-patient variability in EBV-miRNA expression and its molecular significance remain incompletely defined. In this study, small RNA sequencing was performed on 11 primary gastric tumour samples to characterise EBV-derived miRNA expression, followed by quantitative RT-PCR analysis in an extended cohort of 21 tumours for targeted validation. EBV-miRNAs were detected in a subset of tumours and showed marked inter-tumour heterogeneity in abundance. EBV-miRNA-positive tumours were dominated by a conserved set of BART miRNAs, including miR-BART19-3p, miR-BART1-5p, miR-BART10-3p, miR-BART6-3p, miR-BART13-5p, and miR-BART22. These BART miRNAs displayed correlated expression patterns, characterised by concurrent elevation of multiple viral miRNA species within the same tumour samples. To link viral miRNA expression with host molecular responses, in silico virus–host interaction analysis was conducted using ViRBase to prioritise host genes associated with the detected BART miRNAs. *PTEN*, *BCL2L11*, *FOXO3*, and *CDKN1A* were identified as high-confidence targets and selected for experimental assessment. RT-qPCR analysis demonstrated differential expression of these host genes across tumours stratified by EBV BART miRNA abundance. Together, these findings identify a consistent BART miRNA pattern within this cohort. This study provides patient-level molecular evidence linking EBV-miRNA regulatory output to host gene expression states in GC.

## 1. Introduction

GC remains a major global health burden, ranking as the fifth most diagnosed malignancy and the fourth leading cause of cancer-related mortality worldwide, accounting for more than one million new cases and approximately 769,000 deaths annually [1]. Owing to non-specific early symptoms and the lack of reliable early detection biomarkers, a substantial proportion of patients are diagnosed at advanced stages, when curative treatment options are limited, and long-term survival remains poor despite recent therapeutic advances [2]. These clinical challenges highlight the need for improved molecular stratification of GC and the identification of biologically informative markers that can refine diagnosis, prognosis, and personalised treatment strategies.

Comprehensive molecular characterisation by The Cancer Genome Atlas (TCGA) has established four major molecular subtypes of GC, among which EBV-associated GC (EBVaGC) represents approximately 8–10% of cases [3]. EBVaGC is recognised as a distinct biological entity, characterised by lymphoepithelioma-like histology, dense immune cell infiltration, an extreme CpG island methylator phenotype, recurrent *PIK3CA* mutations, and frequent amplification or overexpression of immune checkpoint genes such as *PD-L1* and *PD-L2* [3,4]. On a global scale, EBVaGC contributes substantially to the burden of EBV-driven malignancies, with recent estimates suggesting more than 100,000 new cases annually [5]. These molecular and clinical features indicate that EBV infection plays an active and sustained role in shaping tumour biology and tumour–immune interactions rather than representing a passive passenger event.

A defining molecular feature of EBVaGC is the robust expression of EBV-encoded microRNAs (EBV-miRNAs). EBV encodes approximately 25 precursor miRNAs that give rise to more than 40 mature miRNA species, predominantly derived from the BamHI-A rightward transcript (BART) region, with a limited contribution from the BHRF1 locus [6]. EBV-miRNAs have been shown to regulate multiple cancer-relevant processes, including apoptosis, cell-cycle control, interferon signalling, immune evasion, epithelial–mesenchymal transition, and remodelling of the tumour microenvironment [7,8,9]. In EBVaGC, BART miRNAs are consistently expressed at high levels and have been reported to target tumour suppressor genes, modulate antigen presentation pathways, and promote survival of EBV-infected epithelial cells [10,11]. Their abundance, molecular stability, and viral specificity make EBV-miRNAs attractive candidates for molecular subclassification and biomarker development in EBVaGC.

Despite growing recognition of their biological importance, EBV-miRNA expression patterns in primary gastric tumour tissues remain incompletely characterised. Many published studies have been constrained by limited sample sizes, a predominant focus on host miRNAs, or a lack of validation across independent patient cohorts [12,13,14]. In addition, a substantial proportion of mechanistic insight into EBV-miRNA function has been derived from in vitro model systems, with comparatively limited direct evidence from clinical tumour specimens [5]. Few investigations have adopted integrated analytical approaches that combine unbiased small RNA sequencing with orthogonal validation and cross-cohort comparison to define reproducible EBV-derived miRNA signatures at the patient level.

EBV itself exhibits marked genomic diversity shaped by viral subtype and evolutionary history, with sequence variation influencing viral gene regulation [15,16]. Although EBV strains display geographic clustering at the genomic level, accumulating evidence suggests that EBV-miRNA expression in EBVaGC is largely driven by conserved latency-associated programmes, with sequence variation within the BART region modulating miRNA abundance rather than producing geography-restricted expression profiles [16]. These observations support the existence of shared EBV-miRNA regulatory programmes across diverse patient populations.

Therefore, the present study uses an integrated approach combining small RNA sequencing of gastric tumour tissues and qPCR validation of selected EBV-miRNAs. By doing so, we aim to define reproducible and biologically meaningful EBV-derived miRNA signatures in EBVaGC, thereby advancing mechanistic understanding of EBV-driven gastric carcinogenesis and identifying candidate miRNAs with potential diagnostic and translational relevance.

## 2. Materials and Methods

### 2.1. Ethical Approval

This study was conducted in accordance with the Declaration of Helsinki and approved by the Marmara University Ethics Committee for Non-Interventional and Medical Device-Free Research (approval date: 21 February 2025; protocol no. 09.2025.25-0165).

### 2.2. Patient Selection and Sample Collection

Patients diagnosed with GC and followed at the Department of General Surgery, Marmara University Faculty of Medicine, between 1 January 2024 and 1 July 2024 were evaluated for inclusion in this study. Eligible patients were additionally assessed and underwent hereditary cancer gene panel testing. Written informed consent was obtained from all participants prior to enrolment. Histopathological confirmation of GC was obtained for all patients by board-certified pathologists. EBER in situ hybridisation results were available for a subset of cases and were not used as an inclusion or exclusion criterion. Tumour tissues were then processed for RNA isolation and small RNA sequencing. A total of 21 patients meeting these criteria were enrolled. Peripheral blood samples were collected preoperatively from all patients, and tumour tissue samples were obtained intraoperatively when available. Paired gastric tumour tissue and peripheral blood samples were obtained from 12 patients, while peripheral blood samples alone were collected from 9 patients.

### 2.3. RNA Isolation from Tissue and Plasma

Total RNA, including small RNAs, was isolated from gastric tumour tissues and plasma samples stored at −80 °C. For tissue samples, the miRNeasy Tissue/Cells Advanced Mini Kit (Qiagen, Hilden, Germany) was used according to the manufacturer’s instructions. RNA concentration and purity were assessed using a NanoDrop spectrophotometer (Thermo Fisher Scientific, Hemel Hempstead, UK) by measuring absorbance at 260 nm and 280 nm.

### 2.4. Reverse Transcription and RT-qPCR Analysis

For host gene expression, cDNA was synthesised from total RNA using the QuantiTect Reverse Transcription Kit (Qiagen, Manchester, UK), and RT-qPCR was performed using the QuantiTect SYBR Green PCR Master Mix (Qiagen, Manchester, UK) with the following cycling conditions: 95 °C for 2 min, followed by 40 cycles of 95 °C for 10 s and 60 °C for 60 s. Expression of *PTEN*, *BCL2L11 (BIM)*, *FOXO3*, and *CDKN1A (p21)* was normalised to RPII, and relative expression levels were calculated using the 2^−ΔΔCt^ method.

For EBV-miRNA analysis, cDNA synthesis for miRNA analysis was performed using the miRCURY LNA RT Kit (Qiagen, Manchester, UK), following the manufacturer’s protocol. Quantitative PCR was carried out using the miRCURY LNA SYBR Green PCR Kit (Qiagen, Manchester, UK). Expression levels of EBV BART miRNAs (Table 1), including miR-BART19-3p, miR-BART1-5p, miR-BART10-3p, miR-BART6-3p, miR-BART13-5p, and miR-BART22, were quantified using miRCURY LNA miRNA PCR Assays (Qiagen, Manchester, UK). These candidates were selected based on small RNA sequencing results. RNU6 and SNORD were selected as endogenous controls based on prior EBV-miRNA studies in GC- and EBV-associated epithelial malignancies, in which these controls showed stable expression with minimal Ct variation across tumour tissues and EBV-positive samples [11,13,17]. Thermocycling conditions were as follows: initial denaturation at 95 °C for 2 min, followed by 40 cycles of 95 °C for 10 s and 56 °C for 60 s. Relative miRNA expression was calculated using the 2^−ΔΔC^t method.

### 2.5. Small RNA Sequencing and Bioinformatic Analysis

Library preparation, quality control, and sequencing were performed by Novogene (Cambridge, UK) using the NEB Next Multiplex Small RNA Library Prep Set for Illumina and single-end 50 bp sequencing (SE50) on an Illumina platform. Raw sequencing reads were processed to remove adaptor sequences, low-quality reads, poly-A reads, oversized insertions, and reads without insert tags. Clean reads were analysed using Galaxy software (version 23.1.rc1). Alignment was performed against miRBase version 21 for both hairpin and mature miRNA annotations. miRNA expression levels were normalised as counts per million (CPM), and miRNAs with expression levels below 100 CPM were excluded from downstream analyses. Differential expression was assessed based on normalised read count ratios.

Alignment to EBV genome: Clean reads were aligned to the EBV genome (NC_007605) comprising 171,823 bp circular DNA (VRL 13-AUG-2018) using HiSat (version 2.2.1). Mature miRs were identified and counted using featureCounts (version 2.1.1) using the genomic loci of all 44 EBV-encoded miRs (miRBase) and normalised as counts per million (CPM). Positive control samples were verified using RNAseq data sets from GEO (https://www.ncbi.nlm.nih.gov/geo/, accessed 27 May 2025) including two EBV-positive cell lines: the P3HR1 Burkitt lymphoma line (GSM8684882) and the AGS-EBV-positive gastric cell line (AGS-Akata; GSE302909).

### 2.6. In Silico Virus–Host Target Annotation

In silico virus–host interaction analysis was performed using ViRBase, a curated database that integrates experimentally supported and high-confidence predicted interactions between viral microRNAs and host genes [18]. EBV-derived miRNAs detected in this study were queried against ViRBase to identify putative host target genes. Interaction scores and supporting evidence were used to prioritise candidate targets for downstream analysis and experimental validation. Heatmaps illustrating EBV-miRNA expression patterns across tumour samples were generated using Heatmapper and the R 4.5.2, ggplot2 4.0.0 package.

### 2.7. Statistical Analysis

Statistical analyses were performed using GraphPad Prism version 10.2.2 (GraphPad Software, La Jolla, CA, USA). Data are presented as mean ± standard deviation. One-way analysis of variance followed by Dunnett’s post hoc test was used for comparisons involving miRNA expression levels. Statistical significance was defined as *p* ≤ 0.05.

## 3. Results

**Figure 1:**
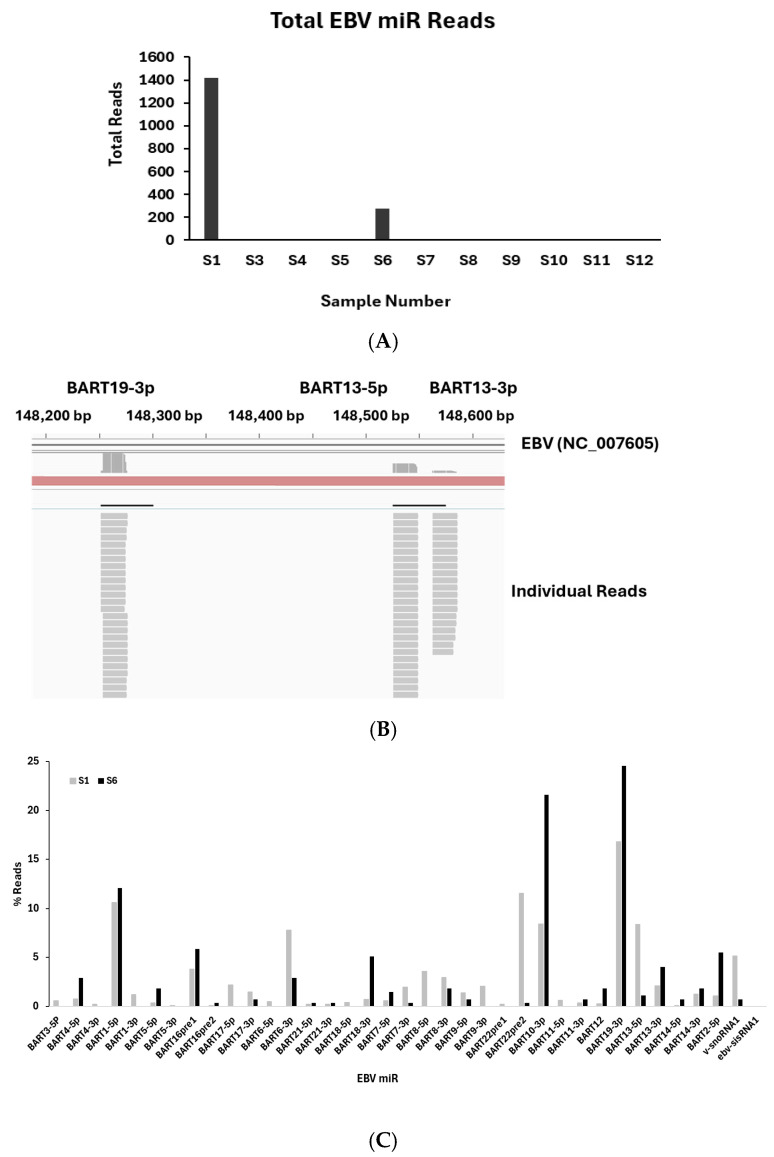
EBV miRNA reads from 12 GC tissue samples. (**A**) Bar graph showing total reads analysed from human gastric RNAseq reads aligned to the EBV (NC_007605) genome. (**B**) Image from Integrative Genomics Viewer showing the reads from the human GC sample (S1) aligned with the EBV genome at BART19 and BART13. (**C**) Reads (%) for each EBV miR in EBV-positive samples (samples S1 & S6).

Small RNA sequencing with alignment to the EBV reference genome revealed heterogeneous expression of EBV-derived viral miRNAs across the GC cohort. EBV-derived reads were detected in a subset of samples and were predominantly concentrated in tumours from two patients, which together accounted for the majority of viral miRNA counts observed (Figure 1A). In these EBV-enriched tumours, the viral miRNA profile was dominated by BART-derived species, with consistently high expression of miR-BART19-3p, miR-BART1-5p, miR-BART6-3p, miR-BART10-3p, miR-BART13-5p, and miR-BART22. Alignment of the human RNAseq reads to the EBV genome showed clean alignment to the mature miR regions (Figure 1B). Although total EBV-miRNA abundance varied between EBV-positive samples, the relative distribution of individual BART miRNAs was comparable, indicating a conserved viral miRNA composition across EBV-enriched tumours (Figure 1C). A similar miR distribution pattern was seen in the AGS-Akata EBV-positive gastric cell line (GSE302909), with high levels of miR-BART6-3p, -10-3p and -19-3p (Figure S1). Targeted RT-qPCR analyses performed in an expanded cohort confirmed the sequencing findings and demonstrated reproducible expression of selected EBV-miRNAs, supporting the robustness and reproducibility of the EBV-miRNA expression pattern identified in this study.

### 3.1. Patient Cohort Characteristics

The clinicopathological characteristics of the GC cohort are summarised in Table 2. The cohort included patients with a range of tumour stages, anatomical localizations, and histological subtypes, encompassing both intestinal and diffuse-type GC. EBER in situ hybridisation results were available for a subset of cases and were used descriptively rather than as an inclusion criterion (Table 2 and Table S1). In this study, EBER status reflects histopathological evidence of viral presence, whereas EBV BART miRNA abundance represents transcriptional activity measured at the molecular level. No single clinicopathological variable demonstrated exclusive segregation with EBV BART miRNA positivity, indicating that detectable viral transcriptional activity was not confined to a specific tumour stage or histological classification.

### 3.2. EBV-miRNA Profiling by Small RNA Sequencing

Small RNA sequencing was conducted on tumour tissues from eleven GC patients. Sample 2 was excluded from analysis because RNA quality did not meet predefined inclusion criteria. EBV-miRNA reads were detected in a subset of tumour samples, with marked inter-sample variability in total viral miRNA abundance. Total EBV-miRNA read counts ranged from 1 to 1421 reads per sample, reflecting heterogeneous EBV transcriptional activity across the cohort (Figure 1). Two samples, S1 and S6, exhibited substantially higher EBV-miRNA loads compared with the remaining tumours. Sample S1 showed the highest EBV-miRNA burden, with 1421 total EBV-aligned reads, while sample S6 displayed intermediate abundance, with 273 reads. All other samples contained fewer than 10 EBV-miRNA reads, consistent with low viral copy number, limited tumour cell infection, and transcriptionally restricted viral states. For classification purposes, EBV BART miRNA status was defined using complementary qualitative and quantitative criteria. EBV BART miRNA-positive denotes detection of at least one EBV BART miRNA by RT-qPCR above the analytical detection threshold, confirmed in technical triplicates following normalisation to the endogenous control. RNA derived from EBV-associated GC tissue was included as a positive control to verify assay performance. EBV BART miRNA-high refers to sequencing-based abundance and was defined as samples in which EBV BART miRNA reads constituted ≥0.001% of total small RNA sequencing reads after adapter trimming, quality filtering, and library normalisation. This threshold provides a platform-independent measure of viral microRNA abundance without altering the relative distribution patterns observed in graphical analyses. Samples not meeting these criteria were designated EBV BART miRNA-low/negative.

These operational definitions were applied uniformly across all downstream analyses and figures to ensure methodological consistency and reproducibility. Across EBV-miRNA-positive samples, the expression profile was characterised by a predominance of miRNAs detected at high read counts. The expression pattern is consistent with a latency I- or latency II-like transcriptional programme typically observed in EBV-associated GC.

### 3.3. Validation of EBV-miRNA Expression by RT-qPCR

**Figure 2:**
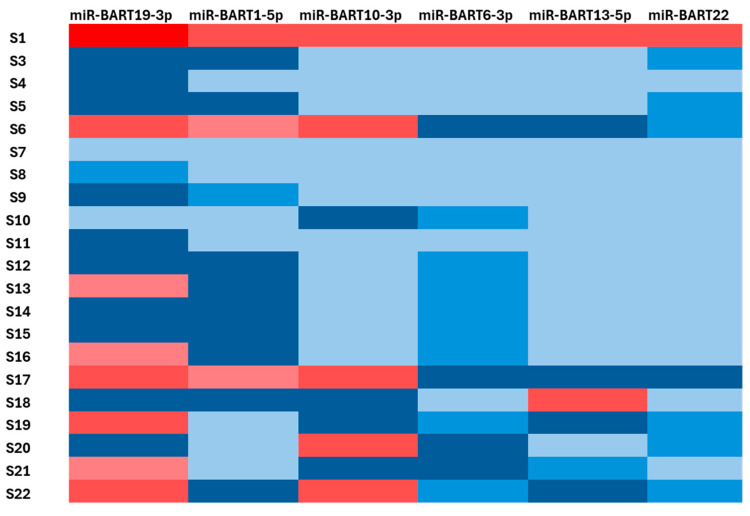
Heatmap of selected EBV BART miRNA expression in GC cohort. Heatmap showing the relative expression of six EBV BART miRNAs with the highest abundance (highest expression level is red; lowest is light blue) in the cohort, demonstrating marked inter-sample heterogeneity and enrichment in a subset of gastric tumours.

RT-qPCR validation was performed for the most abundant EBV BART miRNAs identified by small RNA sequencing, including miR-BART19-3p, miR-BART1-5p, miR-BART10-3p, miR-BART6-3p, miR-BART13-5p, and miR-BART22 (Figure 2). Sequencing-defined EBV-positive tumours showed robust expression of these miRNAs, whereas EBV-low or -negative tumours exhibited low or undetectable levels across the panel. RT-qPCR abundance patterns were concordant with sequencing data, confirming the reproducibility of the viral miRNA profile. Based on concurrent detection of multiple EBV BART miRNAs by RT-qPCR, eight of twenty-one GC samples (38.1%) were classified as EBV-positive, while thirteen samples (61.9%) were classified as EBV-low/negative. EBV-positive samples consistently showed co-expression of miR-BART19-3p and miR-BART10-3p together with additional BART miRNAs, consistent with a latency-associated EBV-miRNA expression profile.

### 3.4. In Silico Identification of EBV BART miRNA Host Targets and qPCR Validation

To identify host genes potentially targeted by EBV BART miRNAs detected in this cohort, virus–host interaction analysis was performed using VirBase [19]. Analysis was restricted to EBV BART miRNAs identified in tumour samples, including BART1-5p, BART6-3p, BART10-3p, BART13-5p, BART19-3p, and BART22.

VirBase analysis identified multiple predicted host targets associated with tumour suppression, apoptosis, and cell-cycle regulation (Table 3). Among these, *PTEN* was identified as a predicted host target of BART19-3p (interaction score 0.9489). *BCL2L11 (BIM)* was identified as a predicted host target of BART1-5p (score 0.963). *FOXO3* and *CDKN1A (p21)* were identified as predicted host targets associated with the detected EBV BART miRNA set, with interaction scores of 0.8899. These targets were selected for downstream expression analysis based on interaction confidence scores and their annotation as regulators of apoptosis and cell-cycle control within VirBase.

**Figure 3:**
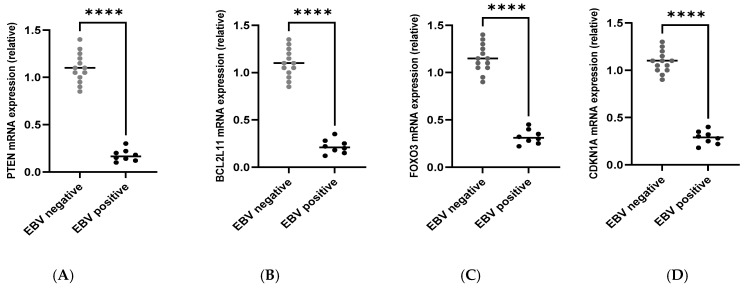
RT-qPCR validation of VirBase-predicted host targets of EBV BART miRNAs in GC. Relative mRNA expression levels of (**A**) *PTEN*, (**B**) *BCL2L11 (BIM)*, (**C**) *FOXO3*, and (**D**) *CDKN1A (p21)* were quantified by RT-qPCR in GC tissues and compared between EBV BART miRNA-positive tumours (*n* = 8, black) and EBV BART miRNA-low/negative tumours (*n* = 13, grey). The Mann–Whitney *U* test was used; **** *p* ≤ 0.0001.

Based on the in-silico target identification, RT-qPCR analysis was performed for *PTEN*, *BCL2L11*, *FOXO3*, and *CDKN1A* in GC samples. Lower expression of *PTEN* in EBV BART miRNA-positive tumours compared with EBV-low/negative samples was detected (Figure 3A). Similarly, *BCL2L11 (BIM)* transcript levels were reduced in EBV BART-positive tumours (Figure 3B). Reduced expression was also observed for *FOXO3* (Figure 3C) and *CDKN1A (p21)* (Figure 3D) in EBV BART-positive samples relative to EBV-low/negative cases.

Across all four targets, inter-sample variability was observed within each group; however, the overall expression patterns consistently differed between EBV BART miRNA-positive and EBV BART miRNA-low/negative tumours (Figure 3). These results confirm differential expression of VirBase-predicted host targets in relation to EBV BART miRNA status in this cohort.

## 4. Discussion

EBV genomic diversity, shaped by viral subtype and evolutionary history, has important implications for understanding strain-dependent regulation of viral gene expression in epithelial malignancies [20]. Large-scale EBV whole-genome sequencing studies demonstrate that the primary axis of EBV diversity is defined by the classical type 1 and type 2 classification, driven largely by sequence divergence within the EBNA2 and EBNA3 loci, with additional sub-structuring among type 1 strains according to geographic origin [15,16]. Principal component analyses of more than 240 EBV genomes show clustering of Asian, European, African, and American isolates; however, these groupings reflect genome-wide patterns of single-nucleotide variation rather than discrete region-specific signatures [16]. Experimental studies further demonstrate that sequence alterations within the BART region can influence the processing efficiency and abundance of EBV-encoded miRNAs, even when mutations occur outside individual hairpin structures, indicating that viral genomic architecture plays a critical role in shaping EBV-miRNA output [21,22]. These features of EBV genomic organisation provide an important framework for interpreting variability in EBV-miRNA output observed across patient tumours in the present study.

EBV is a ubiquitous herpesvirus establishing lifelong latency in more than 90% of the adult population and is implicated in multiple epithelial and lymphoid malignancies [23]. Within GC, EBVaGC is recognised as a distinct molecular subtype defined by viral latency programmes, extensive host DNA methylation, and characteristic somatic alterations, including frequent perturbations of the PI3K pathway [3,24]. These molecular features differentiate EBVaGC from EBV-negative GC and provide a framework for interpreting tumour biology in the present cohort.

In this study of 21 gastric tumours, small RNA sequencing identified a conserved EBV BART miRNA profile that was variably expressed across patients. A core set of BART miRNAs, miR-BART19-3p, miR-BART1-5p, miR-BART10-3p, miR-BART6-3p, miR-BART13-5p, and miR-BART22, dominated the EBV-miRNA landscape in EBV-miRNA-high tumours. These BART miRNAs were concurrently detected at elevated levels within the same tumour samples, indicating a shared expression profile rather than isolated miRNA activation. This multivariate EBV BART signature closely aligns with profiles reported in independent EBVaGC cohorts from East Asia and Europe, in which the same BART miRNAs consistently rank among the most abundant viral small RNAs [11,14,17]. The reproducibility of this core BART set across populations supports the existence of a conserved EBV latency programme characterised by preferential BART output in gastric epithelial contexts.

Notably, EBV-miRNA-high tumours in this cohort were observed across different anatomical locations and Lauren classifications, reinforcing the concept that EBVaGC represents a molecularly defined subgroup rather than a clinicopathologically uniform entity. Importantly, several of the dominant BART miRNAs identified here have been shown in prior experimental studies to converge on pathways directly implicated in GC progression. miR-BART10-3p and miR-BART22 activate canonical Wnt/β-catenin signalling through repression of *DKK1* and *APC*, respectively, thereby enhancing proliferative and invasive phenotypes in EBV-associated epithelial models [25]. miR-BART19-3p further reinforces Wnt signalling and stress-adaptation programmes by targeting *WIF1*, *NLK*, and *GADD45B*, while miR-BART6-3p suppresses innate immune sensing by targeting *DDX58* (RIG-I), attenuating interferon responses and facilitating immune evasion [26,27].

To link viral miRNA profiles with host signalling states in this cohort, in silico virus–host interaction analysis was performed using ViRBase, which integrates experimentally supported and high-confidence predicted miRNA–target relationships [18]. Among the detected BART miRNAs, *PTEN* was annotated as a predicted target of miR-BART19-3p and *BCL2L11* (BIM) as a predicted target of miR-BART1-5p. *FOXO3* and *CDKN1A* were also annotated as predicted host targets connected with the dominant BART species. These genes were selected for RT-qPCR validation to provide direct measurements of host pathway engagement in individual tumours.

The prioritisation of *PTEN*, *FOXO3*, *CDKN1A*, and *BCL2L11* is supported by the extensive literature linking these nodes to molecular hallmarks of EBVaGC. PTEN is a central regulator of PI3K–AKT signalling, and loss of *PTEN* expression has been frequently reported in EBV-positive GC, frequently co-occurring with *PIK3CA* alterations and phosphoinositide pathway activation [3,28]. Methylation-mediated repression of tumour suppressor loci, including the *PTEN* promoter, has been described in EBVaGC and is consistent with reports of widespread epigenetic reprogramming in EBV-associated disease [29]. *FOXO3* and *CDKN1A* integrate stress and DNA damage response signalling, while *BCL2L11* regulates mitochondrial apoptosis. Prior studies have linked EBV-encoded miRNAs to modulation of *BCL-2* family members and apoptotic thresholds in EBV-associated malignancies [30,31].

Crucially, EBV-miRNA abundance varied across patients in this cohort, indicating that the EBV BART signature captures a dimension of molecular EBV activity that is not reflected by binary EBV status alone. This heterogeneity indicates differences in viral regulatory activity between tumours, rather than uniform EBV transcriptional output across EBV-positive cases. The presence of distinct EBV-miRNA expression patterns among EBV-positive tumours allows for finer molecular stratification and facilitates direct linkage between viral regulatory activity and host pathway alterations relevant to tumour progression. This is particularly relevant considering clinical studies reporting higher PD-L1 expression and distinct metastatic behaviour in subsets of EBV-positive GC [32]. In keeping with these findings reported in independent clinical studies, EBVaGC has demonstrated clinically meaningful responses to immune checkpoint inhibition in selected patient cohorts [33]. Host gene interactions were inferred using curated prediction resources and expression concordance analyses; direct experimental confirmation of miRNA–target binding and downstream regulatory mechanisms was beyond the scope of the present study.

Taken together, our study demonstrates that EBV positivity in GC encompasses a reproducible and quantifiable EBV BART miRNA signature at the patient level. Integration of viral miRNA abundance with targeted host gene expression measurements in *PTEN*, *FOXO3*, *CDKN1A*, and *BCL2L11* defines a molecular EBV signature that is tractable in individual tumour specimens. This signature reflects coordinated engagement of PI3K signalling apoptotic regulation, cell-cycle control, and stress-response pathways that are directly relevant to tumour progression and therapeutic susceptibility in EBVaGC.

## 5. Conclusions

By identifying a consistent EBV BART miRNA pattern and relating it to quantifiable host gene expression changes in human tumours, this study provides a patient-level molecular framework for assessing EBV-associated regulatory activity in GC within the limits of cohort size and functional validation. Integration of EBV BART miRNA profiles with biologically relevant host targets indicates their potential utility as functional biomarkers and as indicators of pathway engagement in EBV-associated disease. Future studies in larger cohorts that include EBV genomic variation, host genetic background, and longitudinal clinical follow-up will help clarify the contribution of EBV-derived miRNAs to GC and its progression.

## Figures and Tables

**Table 1:** Primer sequences.

Target	Primer/Assay Sequence (5′–3′)
*RPII*	F: GCACCACGTCCAATGACAT R: GTGCGGCTGCTTCCATAA
*PTEN*	F: ATTGCAGAGTTGCACAATATCC R: CGTCCCTTTCCAGCTTTACA
*BCL2L11 (BIM)*	F: GCCAGCTGCACCTGACGCCCTTC R: CCGCATGCTGGGGCCGTACAGTT
*FOXO3*	F: CACACTACGGCAACCAGACACTC R: TGGGCAGCAAAGGACATCATTGG
*CDKN1A (p21)*	F: CCGTGGACAGTGAGCAGTTGC R: CCCTCCAGCGGCGTCTCC
miR-BART1-5p	F: TCTTAGTGGAAGTGACGTGCTGTG
miR-BART6-3p	F: GCGGGAATCGGACTAGCCTTA
miR-BART10-3p	F: ACTACTAGGAGGAGGAGGAGGA
miR-BART13-5p	F: GTCAGACAGTTTGGTGCTCTAGTTG
miR-BART19-3p	F: GTAACACTTCATGGGTCCCGTAG
miR-BART22	F: GCGTTCAAAGTCGTGGTCTAGTAGT

**Table 2:** Clinicopathological characteristics of the GC patient cohort. Additional clinicopathological variables with partial data availability are presented in Supplementary Table S1.

Patient ID	Sex	Age	TNM	Stage	Tumour Site	Lauren Type	Key Histology
S1	F	44	T4aN0	IIB	Antrum	Diffuse	Adenocarcinoma
S3	F	30	T4aN3a	IIIC	Antrum	Diffuse	Adenocarcinoma
S4	M	70	T4aN3a	IIIC	Antrum/Pyloric region	Mixed	Adenocarcinoma
S5	F	63	NA	NA	NA	NA	Dysplasia
S6	M	44	T3N3a	IIB	Fundus	Diffuse	Adenocarcinoma
S7	M	83	T4aN2	IIIB	Body/Small curvature	Diffuse	Adenocarcinoma
S8	F	65	T1bN0	IA	Body/Greater curvature	Intestinal	Adenocarcinoma
S9	M	60	T1bN0	IA	Body/Small curvature	Intestinal	Adenocarcinoma
S10	M	81	T2N2	IIB	Antrum/Prepyloric	Intestinal	Adenocarcinoma
S11	M	61	T4aN3b	IIIA	Body (Corpus)	Intestinal	Adenocarcinoma
S12	F	62	T3N0	IIA	Antrum/Prepyloric	Intestinal	Adenocarcinoma
S13	M	51	T3N3a	IIIB	Cardia	Intestinal	Adenocarcinoma
S14	M	52	T4aN3b	IIIC	Body/Cardia to small curvature	Intestinal	Adenocarcinoma
S15	F	34	T4aN3b	IIIC	Antrum/Prepyloric	Diffuse	Adenocarcinoma
S16	F	60	T1bN0	IA	Antrum	Intestinal	Adenocarcinoma
S17	M	59	T4aN3M1	IV	Cardia	Mixed	Adenocarcinoma
S18	M	65	T4bN3	IIIC	Body/Small curvature	Mixed	Adenocarcinoma
S19	M	62	M1	IV	Body (Corpus)	Mixed	Adenocarcinoma
S20	M	45	T1bN0	IA	Antrum	Intestinal	Adenocarcinoma
S21	M	89	T3N0	IIA	Antrum/Greater curvature	Intestinal	Adenocarcinoma
S22	M	58	T3N0	IIA	Antrum/Prepyloric	Mixed	Adenocarcinoma

**Table 3:** EBV BART miRNAs detected in this cohort and VirBase-predicted host targets.

EBV BART miRNA	Predicted Host Target Gene	Interaction Type	VirBase Score
BART19-3p	*PTEN*	miRNA–protein	0.9489
BART1-5p	*BCL2L11 (BIM)*	miRNA–mRNA	0.9630
BART1-5p	*FOXO3*	miRNA–mRNA	0.8899
BART1-5p	*CDKN1A (p21)*	miRNA–mRNA	0.8899

## Data Availability

The original contributions presented in this study are included in the article. Further inquiries can be directed to the corresponding author.

## Supplementary Materials

The following supporting information can be downloaded at: https://www.mdpi.com/article/10.3390/v18030329/s1, Figure S1: Relative abundance of EBV BART miRNAs across EBV-positive GC samples. Bar chart showing the percentage of total small RNA sequencing reads attributed to individual EBV BART miRNAs in EBV-positive tumour samples (S1 and S6) and an EBV-positive (+ve) reference RNA control. Reads (%) for each EBV miR in EBV-positive samples; Table S1: Clinicopathological Variables with Partial Data Availability.
