# Supplementary material for: EBV BART microRNA Profiles and Host Gene Links in Gastric Cancer
Source: Viruses. 2026 Mar 7;18(3):329. doi: 10.3390/v18030329 (PMC13030145; doi:10.3390/v18030329)
Supplement: Supplementary file 1 [file viruses-18-00329-s001.zip › viruses-4111164-supplementary.pdf]

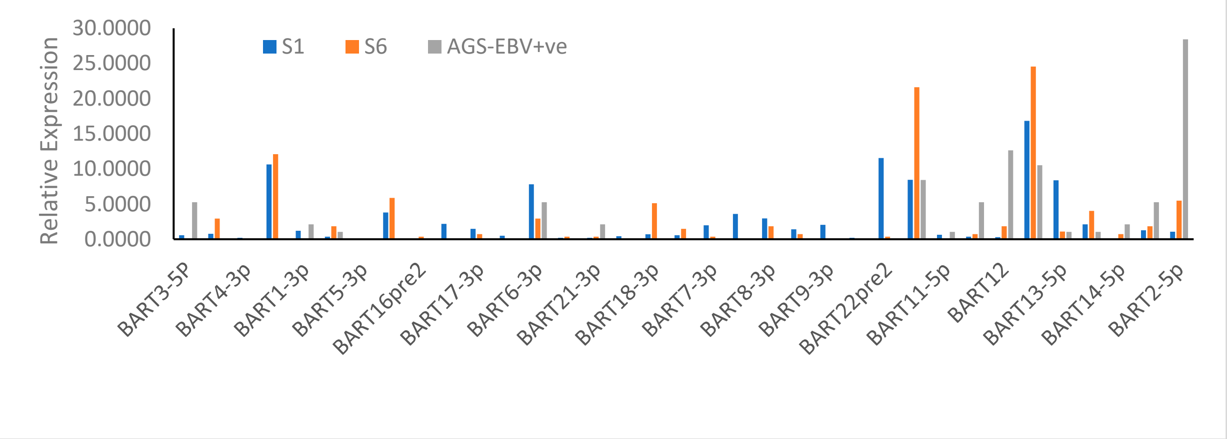

Figure S1: Relative abundance of EBV BART miRNAs across EBV-positive GC samples. Bar chart showing the percentage of total small RNA sequencing reads attributed to individual EBV BART miRNAs in EBV-positive tumour samples (S1 and S6) and an EBV-positive (+ve) reference RNA control. Reads (%) for each EBV miR in EBV-positive samples.

Supplementary Table S1. Clinicopathological variables with partial data availability.

Patient ID	EBER (ISH)	*H. pylori* Status	MMR Status	Neoadjuvant Therapy
S1	Negative	No	Intact	Yes
S3	Positive	Yes	Intact	No
S4	Unknown	No	Intact	Yes
S5	Unknown	Unknown	NA	No
S6	Negative	Yes	Intact	Yes
S7	Negative	No	Intact	No
S8	Negative	Yes	Intact	No
S9	Unknown	No	Intact	NA
S10	Negative	Yes	Intact	No
S11	Negative	Yes	Intact	No
S12	Unknown	No	Intact	No
S13	Unknown	No	Unknown	No
S14	Negative	No	Present	NA
S15	Negative	Yes	Unknown	No
S16	Negative	No	Unknown	No
S17	Negative	Yes	Unknown	No
S18	Negative	No	Unknown	Yes
S19	Unknown	No	Present	NA

S20	Negative	Yes	Unknown	No
S21	Unknown	Unknown	Unknown	No
S22	Negative	No	Present	NA
